# The Dangerous Telephone-Box

**Published:** 1920-07-10

**Authors:** 


### "THE DANGEROUS TELEPHONE-BOX."

FROM A CORRESPONDENT.

*[A few weeks ago (June 5, p. 246) we called attention to the extremely unsatisfactory condition in which one finds the average public telephone-box in London, and our criticisms received a sympathetic hearing from a large number of our readers. We are glad to review the other side of the question, as put forward below and having as its basis an interview with a high official in the London Telephone Service.*—Ed. The Hospital.]

### Past History.

It was in 1905 that Dr. Collingridge, as M.O.H. for the City of London, caused, in view of the popular agitation on the subject, some bacteriological experiments to be carried out, in conjunction with the National Telephone Company, for the purpose of ascertaining whether call-office telephones were likely to be a source of public infection. At that time Professor Klein, who was responsible for the investigation, reported that there was no such risk, and the alarm which had been raised had, apparently, no foundation in fact.

Later, in 1910, as a result of a further outcry, Mr. Samuel (then Postmaster-General) gave instructions that a number of telephones fitted in call-offices should be examined by an expert bacteriologist. This second investigation was carried out by Dr. H. R. D. Spitta. Not only average call-office machines, but also mouthpieces used by known consumptives, were dealt with, and later Dr. Spitta stated that his results had in every respect confirmed Professor Klein's findings in 1905. They showed that the mouthpieces examined were free from tubercle bacilli and diphtheria bacilli, and that no micro-organisms pathogenic to guinea-pigs were present. This did not, however, end the matter, for a further series of telephones, which had been used *solely* by consumptives, were examined, and a second report was submitted, stating the result again to be entirely negative, and Dr. Spitta expressed the opinion that through the medium of the telephone mouthpiece the transmission of tuberculosis was practically impossible. These results, he added, were borne out by independent inquiry initiated by the American Government.

### Local Care of the Boxes.

But let us pass on to the actual process by which these boxes are cared for. In the boxes which are set up in public places the mouthpieces and corresponding parts of the instruments are, by official instructions, washed daily, and the boxes themselves are thoroughly cleaned out and disinfected at least once a week. On the other hand, boxes provided on private property, such as those in shops, are placed there under the care of the owner of that property, and he is responsible for their sanitary condition. The same thing, indeed, might be said of those in a private house. In a vast majority of instances, so the officials not unjustifiably claim, the fault for the general foulness noticeable in boxes during the busy hours of the days lies with the members of the public themselves, and the remedy is 90 per cent., in their own hands. It would be in practice extremely difficult to *enforce* mutual consideration by those who follow in the telephone queue, and much more good might be done by educating the public on the many small but vital ways by which the crowded existence of to-day might gain refinement.

### Size and Ventilation.

The Post Office officials will also point out that, though some of the boxes are small, yet larger ones and more of them are being put up as fast as possible. It is only fair to realise that such changes must occur gradually. In point of fact, the ventilation of the average box is, allowing for the necessity of reasonable exclusion of noise, not so absolutely deficient. All have multiple inlet openings at the floor and corresponding outlets in the roof. All the doors are intended to remain automatically ajar unless they are actually pulled tightly closed by the person inside, and undoubtedly this provision can, with any reasonable interval between calls, allow of a considerable change of the air inside. But, again, if there is *no* interval it is more than difficult to see how fresh air as from the hillsides can be introduced within thirty seconds to the interior of any box of reasonable dimensions. That the authorities are improving, extending, and modernising the whole of the system and its essential instruments is a statement in the truth of which the writer has every confidence after hearing the official explanations. The telephone service has always possessed a huge army of critics. Their outcries have always been assured of finding many sympathetic ears, but it is probable that but a tithe of the accusations are fully justified. An inspection of the early pages of a telephone directory with its simple "How to use the Telephone Service: Some Suggestions," will bring to mind ample illustrations of the many ways in which the public so often fails to appreciate the officials' side of the telephone question.

